# Novel Drugs in a Pipeline for Progressive Multiple Sclerosis

**DOI:** 10.3390/jcm11123342

**Published:** 2022-06-10

**Authors:** Klaudia Sapko, Anna Jamroz-Wiśniewska, Konrad Rejdak

**Affiliations:** Department of Neurology, Medical University of Lublin, 20-059 Lublin, Poland; anna.jamroz-wisniewska@umlub.pl (A.J.-W.); konrad.rejdak@umlub.pl (K.R.)

**Keywords:** multiple sclerosis, progressive multiple sclerosis, neuroprotective drugs, remyelinating drugs, clinical trials

## Abstract

Multiple sclerosis (MS) is a widely known inflammatory, demyelinating disease of the central nervous system. The pathogenesis of progressive multiple sclerosis (PMS) is a complex, multi-level process that causes therapeutic difficulties. Along with variables such as age and duration of the disease, pathogenetic mechanisms change from inflammatory to neurodegenerative processes. Therefore, the efficacy of available anti-inflammatory drugs approved for the treatment of PMS, such as ocrelizumab or siponimod, is limited in time. In search of innovative solutions, several research studies have been conducted to evaluate the effectiveness of drugs with neuroprotective or remyelinating effects in PMS, including biotin, ibudilast, simvastatin, alpha-lipoic acid, clemastine, amiloride, fluoxetine, riluzole, masitinib, opicinumab, and lamotrigine. The current review includes those compounds, which have entered the clinical phase of assessment, and the authors discuss future prospects for successful PMS treatment.

## 1. Introduction

Multiple sclerosis (MS) is a chronic inflammatory demyelinating disease that mainly affects people aged 20–40 years who live in temperate climate areas in the northern hemisphere. MS is one of the leading causes of disability in young adults [1,2].

Traditionally, the following three phenotypes of the disease are distinguished: relapsing-remitting MS (RRMS), primary progressive MS (PPMS), and secondary-progressive MS (SPMS). Nevertheless, in the new classification of MS, PPMS and SPMS are defined as one unified progressive MS (PMS) type, which then can be divided into active or inactive, and progressive or non-progressive [3].

The clinical feature of PMS is a gradual increase in disability, but the pathological mechanisms underlying this process are different from those in RRMS. In PMS, disease mechanisms initially change with age and duration of the disease, from inflammatory to predominant neurodegenerative processes. Other PMS-specific neuropathological mechanisms include cortical demyelination, formation of B lymphocyte lymphoid aggregates in the meninges, and demyelination of the gray matter in the deep gray matter nuclei, the cerebellum, and the spinal cord [4,5]. Chronic active lesions, characterized by remyelination and degeneration of axons with peripheral inflammatory demyelination, are also a feature of MS pathology progression. These are non-gadolinium-enhancing lesions with paramagnetic rims visible in magnetic resonance imaging (MRI), which indicate an aggressive course of the disease and extensive tissue damage [6,7]. On the other hand, the conducted functional neurophysiological studies emphasize the importance of intracortical sensorimotor networks in monitoring the development of disability through the observation of hand dexterity impairment in PMS [8,9,10].

## 2. Time as a Key Element in the Treatment of PMS

There are three classes of drugs used for the treatment of MS, including steroids reserved for relapse treatment, symptomatic drugs, and continuously researched disease-modifying drugs (DMDs) used to stop disease progression.

The currently available drugs approved for the treatment of PMS, such as siponimod (SPMS), which is a small molecule that crosses the blood-brain barrier, or monoclonal antibody—ocrelizumab (PPMS), are anti-inflammatory drugs [11,12]. The decision to initiate such treatment should be based on central nervous system (CNS) inflammation assessed by clinical and MRI activity, along with biomarker assessment. However, anti-inflammatory treatment must be initiated early in PMS when inflammation in the CNS is the predominant process, as was found in a multicenter study with Ocrelizumab, which confirmed that early initiation of this drug has a positive effect on modification of disability in MS patients. [13,14]. In contrast, neuroprotective and remyelinating drugs may have a wider window of opportunity as they might inhibit apoptosis, induce neurotrophic activity, stimulate defense mechanisms based on antioxidant activities, or prevent oxidative damage.

In recent years, research has been carried out on the validity of the use of new substances such as biotin, ibudilast, simvastatin, alpha-lipoic acid, clemastine, amiloride, fluoxetine, riluzole, masitinib, opicinumab, and lamotrigine in the treatment of PMS [15].

## 3. New Potential Drugs in the Treatment of PMS

### 3.1. Biotin

Biotin, otherwise referred to as vitamin B_7_;, is an essential cofactor for five carboxylases involved in the production of energy and fatty acids. It displays good bioavailability, fast absorption, and excretion [16,17]. Biotin may reduce axonal hypoxia and promote axonal remyelination through enhanced energy production [18].

The first open-label, unblinded pilot study included 23 patients with PMS. Biotin administered orally at an increased dose of 300 mg/day for a maximum of 36 months showed encouraging efficiency in PMS treatment. Some degree of clinical improvement was reported by over 90% of the patients, including a 22% Expanded Disability Status Scale (EDSS) reduction, visual acuity improvement, magnetic resonance (MR) spectroscopy, P100 latency on visual evoked potentials (VEPs), neurological symptoms, and clinical examination [19].

The next randomized, double-blinded, placebo-controlled study called MS-SPI included 154 patients aged 18–75 years with PMS, who were randomized to oral biotin treatment at a dose of 300 mg/day or placebo in a 2:1 ratio for 12 months. The primary endpoint, that is at least a one-point EDSS decrease or at least a 20% decrease in Timed 25-Foot Walk (T25FW) at month 9, confirmed at month 12, was met in 13 (12.6%) patients treated with biotin versus none of the patients treated with placebo. Nevertheless, new T2 lesions occurred in 23.4% of the biotin-treated patients and 13.0% of the placebo-treated ones. The analysis showed that the patients with lower EDSS were more likely to pass the primary endpoint. Generally, biotin at a high dose was well tolerated by the patients [20].

Another open-label trial included 33 patients with PMS and 10 patients with RRMS and was designed to evaluate the influence of biotin on the clinical condition and magnetic resonance imaging (MRI) in MS patients. The patients received 300 mg of biotin per day for 12 months and had laboratory tests and EDSS assessed every 3 months, and MRI performed at the start and after 1 year [21]. Despite all this, no benefits were observed in the study. However, the mean age of the patients was higher (61 years) compared to those involved in the previous study (average age: 50.7–52.8 years), where biotin was found to be positive [19].

In recent years, another randomized, double-blind, parallel-group, placebo-controlled SPI2 study was conducted to determine the safety and efficacy of biotin in PMS. The study included 642 patients aged 18–65 years who were randomly assigned (1:1) to receive oral biotin 100 mg three times daily or a placebo. The comparison of the proportion of patients with improvement in EDSS or TW25 at month 12, confirmed at month 15, versus baseline was the primary endpoint. The results showed that, for the primary outcome, improvement was achieved by 39 (12%) of 326 patients in the biotin group and 29 (9%) of 316 in the placebo group. The study revealed that biotin did not significantly improve the disability or walking speed of PMS patients [22].

On the other hand, a retrospective multicenter French study of 2628 PMS patients compared to a control group of 654 patients was conducted to analyze the relapse rate during biotin therapy. Even though the number of relapses in patients without previous inflammatory activity gradually increased while taking biotin, the results of the study excluded the possibility of an increased risk of relapses during biotin therapy [23].

In preliminary studies, high-dose biotin was considered a promising drug for the treatment of PMS, but a recent SPI2 study showed that biotin did not significantly affect the patient’s disability, which is the primary therapeutic target. Moreover, though biotin is believed to be safe and well-tolerated by patients, it may interact with laboratory testing of biotinylated assays. Thus, the doctor or the technician performing the test should be informed about the patient’s intake of biotin, especially in high doses, to correctly interpret the test [24,25].

### 3.2. Ibudilast

Ibudilast is a small molecule that inhibits the macrophage migration factor (MIF), toll-like-receptor-4 (TLR4), phosphodiesterase-4 (PDE-4), and phosphodiesterase-10 (PDE-10), as well as suppresses the production of tumor necrosis factor-α (TNF- α), and pro-inflammatory interleukin IL-1β and IL-6 [26,27]. Ibudilast crosses the blood-brain barrier and has a neuroprotective effect resulting from the inhibition of the production of IL-10 and neuronal cell death induced by microglial activation [28,29].

A phase II double-blinded clinical trial called NeuroNEXT 102 (NN102) or Secondary and Primary Progressive Ibudilast NeuroNEXT Trial in Multiple Sclerosis (SPRINT-MS) was conducted to investigate the effects of ibudilast on PMS patients. The study involved 255 PPMS and SPMS patients, randomized 1:1, with a median age of 56 years and a disease duration of 11 years; 129 patients received ibudilast orally up to 100 mg, and 126 patients received a placebo for 96 weeks. The primary endpoint, change in the brain parenchymal fraction, was reduced by 48% in the patients taking ibudilast compared to those taking placebo. However, there was a higher incidence of gastrointestinal disorders in the patients treated with ibudilast [30,31].

The first results of studies with ibudilast in PMS are promising, but larger observational studies are needed to determine the effectiveness and safety of its use in PMS.

### 3.3. Simvastatin

Simvastatin is a 3-hydroxy-3-methyl-glutaryl-CoA reductase (HMG-CoA) inhibitor and is used to treat hyperlipidemia, usually in high-risk patients with coronary artery disease. The mechanism of action of simvastatin in MS is based on the decrease of T-cell proliferation and inhibition of the presentation of major histocompatibility complex (MHC) class II antigen, production of anti-inflammatory Th2 lymphocytes, a decrease in adhesion molecule expression, and a protective role for cells [32,33].

A phase II double-blinded, placebo-controlled clinical trial called MS-STAT was performed to assess the effect of administration of simvastatin in high doses on brain atrophy in 140 SPMS patients. The patients were randomized 1:1 and received 80 mg of simvastatin per day or a placebo. The study showed a 43% reduction in the mean annualized rate of brain atrophy and a mean change in the EDSS score in the patients taking simvastatin versus those taking placebo. No effect of simvastatin on some secondary outcomes, such as the relapse rate and immunological markers, and new or enlarging T2-hyperintense lesions on MRI, was found. The incidence of adverse effects was approximate in both groups [34].

The reanalysis of the MS-STAT study for the impact of simvastatin on neuropsychiatric, cognitive, and health-related quality-of-life measures in SPMS revealed a positive influence of this drug on frontal lobe function and the physical measure of quality of life. Simvastatin had no effect on the other factors, although these potential effects show how important it is to assess the quality of life in PMS studies [35]. Simvastatin is a promising drug and the subject of current research. At present, the effect of simvastatin on vascular perfusion and oxidative damage, and on slowing the progression of disability in patients with SPMS, is still under investigation (Table 1).

### 3.4. Alpha-Lipoic Acid

Alpha-lipoic acid (ALA) is an endogenous antioxidant synthesized naturally in the liver or provided by daily food products. It is believed that it may have a neuroprotective effect on MS patients through the downregulation of inflammatory cytokines, the repair of oxidative damage, T-cell infiltration into the CNS, and metal chelation [36].

The effect of ALA on MS has been the subject of several pilot studies revealing that ALA impacts positively on MS activity and T-cell migration by reducing the soluble intercellular adhesion molecule-1 (sICAM-1) and matrix metallopeptidase 9 (MMP-9) factors [37]. It also reduces interferon-γ (INF-γ), transforming growth factor-β (TGF-β), IL-4 levels, and oxidative stress [38,39].

Ultimately, the impact of ALA on patients with SPMS was studied in the phase II randomized, double-blinded, placebo-controlled clinical trial. ALA, at a dose of 1200 mg/day, was administered to 51 patients for 2 years. The results showed a 68% reduction in brain atrophy in the patients treated with ALA compared to placebo. No differences between the ALA and placebo patients in the clinical outcomes, brain substructures, and optical coherence tomography metrics were observed. Overall, ALA was well tolerated during the study, except for a higher percentage of gastrointestinal complaints compared to placebo-treated patients. It seems that ALA is a promising drug that could be used in the prevention of brain atrophy in MS patients [40]. Research is currently underway on the effect of ALA on the progression of disability in patients with PMS (Table 1).

### 3.5. Clemastine

Clemastine is an antagonist of H1 and an M1/M3 receptor with antihistaminic activity. In the pathogenesis of multiple sclerosis, clemastine probably differentiates oligodendrocytes and remyelination depending on the M1 muscarinic receptor [41,42].

The effectiveness of clemastine fumarate in MS patients with chronic optic neuropathy has been confirmed in a phase II double-blinded, placebo-controlled, cross-over trial called ReBUILD. The study included 50 patients who received 5.3 mg of clemastine fumarate orally for 90 days, followed by a placebo for 60 days, or vice versa, and it lasted 150 days. The patients were periodically evolved by examining VEPs. The shortening of the P100 time delay in VEPs for potentials with a full field of view was observed. This study was the first to assess the effectiveness of remyelinating drugs in chronic demyelinating lesions, and the positive results of this study led to the search for new drugs with remyelinating effects [43]. For now, the effectiveness of clemastine as a remyelinating agent in acute optic neuritis is currently being tested (Table 1).

### 3.6. Amiloride, Fluoxetine, and Riluzole

Another three potential neuroprotective drugs, amiloride, fluoxetine, and riluzole, were tested against a placebo in a randomized, multiarm, double-blinded trial called MS-SMART [44]. Amiloride is a diuretic, and fluoxetine is a selective serotonin reuptake inhibitor [45]. Riluzole is an inhibitor of the sodium channel and has anti-glutamatergic properties; it is the only disease-modifying drug registered by the European Medicine Agency (EMA) to treat amyotrophic lateral sclerosis (ALS), but its efficiency is questionable [46]. However, a preliminary one-step clinical trial with 16 SPMS patients receiving 50 mg of riluzole for a year revealed that it may reduce T1 hypointense lesion accumulation in the cervical spine and brain atrophy. Nevertheless, in the MS-SMART study involving 445 patients with SPMS (amiloride—111 patients, fluoxetine—111, riluzole—111, and placebo—112), no difference was observed in the percentage of brain volume change or new T2 lesions [47]. Ultimately, amiloride, fluoxetine, and riluzole turned out to be ineffective in PMS treatment.

### 3.7. Masitinib

Masitinib is a selective tyrosine kinase inhibitor that effectively inhibits the migration, survival, and activity of mast cells [48,49]. The effectiveness of its use in PMS was initially confirmed in a multicenter, randomized, placebo-controlled, proof-of-concept trial. The study included 35 patients randomized to receive masitinib orally at 3–6 mg/kg/day for at least 12 months or a placebo. The results showed a non-statistically significant difference in the MS-related impairment in PMS patients, but there was no difference in the EDSS score [50]. A phase III clinical trial with masitinib involving patients with PPMS and SPMS was conducted and was due to end in September 2020, but the results are not known yet [51]. Initial studies with masitinib did not produce the expected results, but it is possible that the drug was more effective in phase III of the trial.

### 3.8. Opicinumab

Opicinumab is a monoclonal antibody directed at a leucine-rich repeat and immunoglobulin domain-containing Nogo receptor-interacting protein 1 (LINGO-1). Opicinumab was a promising candidate for the treatment of MS as it showed some remyelinating effects in animal models of demyelination [52]. Although treatment with opicinumab showed a moderate remyelinating effect in patients with acute optic neuritis, the drug failed in a phase II clinical trial called SYNERGY that included active SPMS patients [53,54].

### 3.9. Lamotrigine

Lamotrigine is a blocker of the sodium channel. Its effectiveness in MS treatment was studied in a trial involving 120 patients with SPMS, randomized to lamotrigine 400 mg/day orally or placebo, administered for 2 years. Unfortunately, there was no difference between the two groups in the mean change in cerebral volume per year. Lamotrigine reduced deterioration in the T25FW test but did not affect other secondary clinical outcome measures [55]. Preliminary research results show that lamotrigine has no significant positive effects in treating MS.

## 4. Future Perspectives

There is currently a lot of research going on into new potential compounds that could be used in the treatment of PMS.

Therapy using autologous hematopoietic stem cell transplantation (AHSCT) in MS has emerged as a treatment option almost entirely based on empirical evidence from observational studies [56,57]. However, its benefits are limited because of the risk of side effects, especially infections or treatment-related mortality. That is why, in the current guidelines, AHSCT is considered an experimental treatment option for younger patients with insufficient response to regular high-effective DMDs and high inflammatory activity [58].

In addition, mesenchymal stem cells (MSC) therapy is taken into consideration in the PMS because of its neuroprotective and repair-promoting functions [59]. So far, some studies of MSC transplantation in PMS have been conducted [60,61]. An assessment of the Bone Marrow-derived Cellular Therapy in Progressive MS (ACTiMuS) trial aimed at a more in-depth analysis of the effectiveness of autologous bone marrow intravenous infusion without myeloablation in PMS has also been started [48].

Another promising method of PMS treatment is ATA188, an advanced cell therapy for the elimination of EBV-infected cells. This method involves sensitizing HLA-matched donor T cells against EBV antigens and administering them to recipients. The first results of two small safety trials of this method in patients with PMS have already been presented [62,63].

Last but not least, combination therapy should be seriously considered when planning future clinical trials. It seems unlikely that any single drug would stop the complex pathogenetic process in MS. In order to achieve that goal, we should act on different levels of the cascade, inhibiting demyelination and neuronal death and, at the same time, promoting remyelination and structural restoration processes.

## 5. Conclusions

The treatment of PMS presents a major challenge due to the complex pathogenesis of this process. It is believed that PMS disease mechanisms change from initially inflammatory to predominantly neurodegenerative processes with age and duration of the disease. Therefore, the therapeutic window for the use of anti-inflammatory drugs is very narrow, giving more possibilities to drugs with neuroprotective and remyelinating properties. Recently, a lot of research into the spectrum of mainly neuroprotective (ibudilast, simvastatin, alpha-lipoic acid, amiloride, fluoxetine, riluzol, masitinib) and a few remyelinating drugs (i.e., biotin, clemastine, opicinumab) that can be used to treat PMS has been conducted (Table 2). While preliminary results of the research on some of these drugs are very promising, the effectiveness and safety of these therapies still need to be confirmed by detailed studies. A combination therapy acting on different levels of the pathological cascade and initiated early in the disease process seems to constitute a more promising and pragmatic approach for future clinical trials.

## Figures and Tables

**Table 1:** A summary of ongoing clinical drug trials for the treatment of progressive multiple sclerosis. (Website: https://www.clinicaltrials.gov/; accessed on 12 April 2022.).

Trial	Intervention	Primary Outcome Measures	Secondary Outcome Measures
A Randomized, Double-blinded, Placebo-controlled Single-site Study of High Dose Simvastatin Treatment for Secondary Progressive Multiple Sclerosis: Impact on Vascular Perfusion and Oxidative Damage	Simvastatin 40 mg for first 4 weeks 80 mg (if tolerated) thereafter up to 17 weeks	Effect on cerebral blood flow	MRI: glutamate levels Adaptive Optics Scanning Laser Ophthalmoscope measurements of blood flow MRI: arterial spin labeling measurements of blood flow MRI: brain atrophy Clinical Outcome: EDSS
A Phase III Randomized, Double-Blinded Clinical Trial Investigating the Effectiveness of Repurposed Simvastatin, Compared to Placebo, in Secondary Progressive Multiple Sclerosis, in Slowing the Progression of Disability	Simvastatin One (1 = 40 mg) tablet/day at night, for 1 month Two (2 = 80 mg) tablets/day at night, for the next 35 months Placebo Equivalent placebo	Time to the confirmed disability progression between simvastatin and the placebo arm based on changes in the EDSS scores compared to baseline	Response rate on the patient reported outcome form Multiple Sclerosis Walking Scale-12 version 2, Impact Scale-29 version 2 Cost effectiveness of intervention Change in the time taken to complete timed 25-foot walk Change in the time taken to complete the9 hole peg test Evaluating changes in the degree of disability based on the modified Rankin Scale Change in frontal lobe function based on frontal assessment battery scores Difference in the number and severity of multiple sclerosis-related relapse events between the treatment groups
Lipoic Acid for the Treatment of Progressive Multiple Sclerosis	Lipoic acid 200 mg/day for 2 years Placebo taken daily for 2 years	Mobility: timed 25-foot walk	Mobility: timed 2-min walk Mobility: fall count Brain atrophy by MRI
Assessment of Clemastine Fumarate as a Remyelinating Agent in Acute Optic Neuritis (ReCOVER)	Clemastine 12 mg (4 mg 3×/day) for 7 days followed by 8 mg (4 mg 2×/day) until 3 months Placebo Equivalent placebo	Change in P100 latency on full-field visual evoked potential Change in low contrast visual acuity	Change in retinal nerve fiber layer thickness on optical coherence tomography Radiological outcomes assessed by magnetic resonance imaging EDSS score

MRI—magnetic resonance imaging; EDSS—expanded disability status scale.

**Table 2:** A summary of completed clinical trials in patients with progressive multiple sclerosis.

Study	Treatment Method	Number of Patients and Duration	Results
Sedel (2015) [19]	Biotin 300 mg/day	23 PMS patients 2–36 months	Positive
Tourbah (2016) [20]	Biotin 300 mg/day compared to placebo	154 PMS patients 12 months	Positive on EDSS/T25FW Negative on MRI
Birnbaum (2017) [21]	Biotin 300 mg/day	33 PMS and 10 RRMS patients 12 months	Negative, but older patients
Cree (2020) [22]	Biotin 300 mg/day compared to placebo	642 PMS patients 15 months	Negative
Fox (2016, 2018) [30,31]	Ibudilast 100 mg/day compared to placebo	255 PPMS and SPMS patients 96 weeks	Positive, but higher incidence of gastrointestinal disorders
Chataway (2014) [34]	Simvastatin 80 mg/day compared to placebo	140 SPMS patients	Positive on brain atrophy and EDSS No effect on the relapse rate, immunological markers, and MRI
Spain (2017) [40]	Alpha-lipoic acid 1200 mg/day compared to placebo	51 SPMS patients 2 years	Positive on brain atrophy No effect on the clinical outcomes, brain substructures and optical coherence tomography metrics
Green (2017) [43]	Clemastine fumarate 5.3 mg/day compared to placebo	50 MS patients 150 days	Positive on the P100 time delay in VEPs
Vermersch (2012) [50]	Masitinib 3–6 mg/day compared to placebo	35 PMS patients 12 months	Negative
Kapoor (2010) [55]	Lamotrigine 400 mg/day compared to placebo	120 SPMS patients 2 years	Positive on T25FW Negative on the mean change in cerebral volume

PMS—progressive multiple sclerosis; RRMS—relapsing-remitting multiple sclerosis; PPMS—primary progressive multiple sclerosis; SPMS—secondary progressive multiple sclerosis; MS—multiple sclerosis; EDSS—expanded disability status scale; T25FW—timed 25-foot walk; MRI—magnetic resonance imaging; VEPs—visual evoked potentials.

## Data Availability

Not applicable.
